# Editorial: thematic issue on Polar and Alpine Microbiology

**DOI:** 10.1093/femsec/fiae030

**Published:** 2024-03-22

**Authors:** Liane G Benning, Dirk Wagner, Catherine Larose, Nina Gunde-Cimerman, Max M Häggblom

**Affiliations:** German Research Centre for Geosciences GFZ, Telegrafenberg A71-359, 14473 Potsdam, Germany; German Research Centre for Geosciences GFZ, Telegrafenberg A71-359, 14473 Potsdam, Germany; Université Grenoble Alpes, CNRS, Institute of Geosciences of the Environment IGE, CS 40700, 38 058 Grenoble, France; University of Ljubljana, Department of Biology, Biotechnical faculty, Jamnikarjeva 101, 1000 Ljubljana, Slovenia; Rutgers University, Department of Biochemistry and Microbiology, 76 Lipman Drive, New Brunswick, NJ 08901-8525, United States

Microorganisms, representing all domains of life, have successfully colonized Earth’s cold habitats. This thematic issue brings a focus on the microbial ecology of the cryobiosphere. Knowledge of biodiversity and functional roles of microorganisms inhabiting these cold environments is essential to our understanding of polar and alpine ecosystem processes in a changing climate.

The 9th International Conference on Polar and Alpine Microbiology (PAM22) took place in October 2022 in Potsdam, Germany, following a 3-year COVID driven hiatus. The decision to postpone was made in late 2020 because the strength and success of PAM meetings have always been relying on in-person interactions between participants, which are more difficult online. The meeting attracted more than 120 participants from 21 different countries, of which 41% were student participants. The science spanned nine topics that addressed a wide range of research areas from: “Microbial communities and global change” and “Microbial gene pool and biotechnology,” “Eukaryotic microbial diversity” and “Adaptation, survival and subzero activity,” and all the way to “Carbon and nitrogen turnover” and “Plant–microbe interactions,” as well as an open session on “Cryosphere Microbiology.” The 105 submitted abstracts were presented during the week-long meeting either as talks (60%), including nine keynote presentations, or as posters (40%). We placed a big emphasis on promoting early career scientists for talks and to chair sessions during the meeting. At the end of the PAM22 conference a call was issued for manuscripts to be assembled in a virtual *FEMS Microbiology Ecology* Thematic issue. This thematic issue on Polar and Alpine Microbiology, continuing earlier thematic issues that have focused on the microbial ecology of Earth’s cryobiosphere, encompasses 30 peer-reviewed papers, with the majority being based on research presented at the PAM22 conference. We have also attracted other submissions in the field, thus broadening the scope even further.

The articles detail field studies that span all three poles with most covering microbial related research either from Arctic regions (10 papers), various mountain regions (from the Himalayas and Tibet to the US Cascades and the European Alps; 10 papers) and also Antarctica (five papers). A few papers also address comparisons between Alpine and Arctic processes or detail laboratory studies targeting adaptations or sample analyses issues (e.g. Broadwell et al. 2023).

The plethora of studies on Arctic microbial ecosystems cover aspects from the top of glaciers all the way to the marine realm. These include microbial studies related to tundra (Almela et al. 2023, Doherty et al. 2023, Michaud et al. 2023, Touchette et al. 2023), proglacial (Luláková et al. 2023, Poppeliers et al. 2022, Masumoto et al. 2023) or abruptly thawed permafrost soils (Scheel et al. 2023), to seasonal microbial reactions that control land–ocean connectivity in tidal flats (Handler et al. 2024), and all the way to processes in coastal and open Arctic waters (Robicheau et al. 2023, von Friesen et al. 2023b) and even cover coastal thermokarst lake communities (Yang et al. 2024). Complementing these are a variety of papers that address supraglacial diversity in snow and ice habitats in Greenland, Svalbard, and Norway (Jaarsma et al. 2023, Sanchez-Cid et al. 2023, Suzuki et al. 2023) or compare Alpine and Arctic processes either addressing best practices in sequencing for snow and ice algal communities (Remias et al. 2023) or assessing how vegetation controls microbial responses to drought (Fry et al. 2023). Noteworthy, here is the Jaarsma et al. (2023) paper that was selected as one of seven FEMS Journals Article Awards for 2023 (https://fems-microbiology.org/about_fems/network-and-activities/awards/article-awards/).

Studies that address snow, ice, and soil microbial processes in mountain regions report on the controls of snow algae blooms (Hamilton and Having 2023, van Hees et al. 2023), plant–fungal symbionts (Hiiesalu et al. 2023), or bacterial communities in proglacier settings (Mukhia et al. 2024) to microbial successions in chronosequences of proglacier soils and thawing permafrost soils in Tibet (Khan et al. 2023, Tang et al. 2023) and all the way to microsymbionts modulating plant characteristics in the High Atlas mountains of Morocco (Lamrabet et al. 2023) or antibiotic-producing Streptomyces in Himalayan soils (Bhat et al. 2024).

Work that highlight research results from Antarctica includes evaluations of benthic bacterial and diatom communities in lakes (Kollár et al. 2023), to fungal roles in endolithic communities (Biagioli et al. 2023) to bacterial roles in Antarctic lichens (Woltyńska et al. 2023) and naturally work in soils linked to microbial succession in proglacial soils (Vimercati et al. 2022).

The papers of this thematic issue highlight the vibrant Polar and Alpine Microbiology research community. Many of the papers invariably address issues that are of high relevance in the currently fast changing climate. It is clear that advances in sequencing and analytical opportunities allow us more and more to quantify important microbiological and microbial ecological processes that affect and are in turn affected by polar and alpine processes.

We dedicate this issue to our good friend and wonderful colleague, S. Craig Cary, who passed away in February 2024. He was a pioneering and inspiring researcher in the study of microbial life in extreme environments, including the cryobiosphere. He spent several seasons in Antarctica to study the physiology, biochemistry, and ecology of microbial communities in polar deserts. Craig was an active participant in the Polar and Alpine Microbiology Conference series, hosting the 2019 meeting at the University of Waikato—Te Whare Wānanga o Waikato, Hamilton, New Zealand (Cary et al. 2020). He will be missed by all of us.
